# Assessment of fitting methods and variability of IVIM parameters in muscles of the lumbar spine at rest

**DOI:** 10.3389/fmscd.2024.1386276

**Published:** 2024-04-10

**Authors:** Erin K. Englund, David B. Berry, John J. Behun, Lawrence R. Frank, Samuel R. Ward, Bahar Shahidi

**Affiliations:** 1 Orthopaedic Surgery, University of California, San Diego, La Jolla, CA, United States; 2 Radiology, University of Colorado Anschutz Medical Campus, Aurora, CO, United States; 3 Radiology, University of California, San Diego, La Jolla, CA, United States; 4 Orthopaedic Surgery, Radiology, Bioengineering, University of California, San Diego, La Jolla, CA, United States

**Keywords:** IVIM, skeletal muscle, intravoxel incoherent diffusion-weighted imaging, MRI, spine

## Abstract

Intravoxel incoherent motion (IVIM) MRI provides insight into tissue diffusion and perfusion. Here, estimates of perfusion fraction (f), pseudo-diffusion coefficient ($D^{*}$), and diffusion coefficient (D) obtained via different fitting methods are compared to ascertain (1) the optimal analysis strategy for muscles of the lumbar spine and (2) repeatability of IVIM parameters in skeletal muscle at rest. Diffusion-weighted images were acquired in the lumbar spine at rest in 15 healthy participants. Data were fit to the bi-exponential IVIM model to estimate $f,\;D^{*}$ and D using three variably segmented approaches based on non-linear least squares fitting, and a Bayesian fitting method. Assuming that perfusion and diffusion are temporally stable in skeletal muscle at rest, and spatially uniform within a spinal segment, the optimal analysis strategy was determined as the approach with the lowest temporal or spatial variation and smallest residual between measured and fit data. Inter-session repeatability of IVIM parameters was evaluated in a subset of 11 people. Finally, simulated IVIM signal at varying signal to noise ratio were evaluated to understand precision and bias. Experimental results showed that IVIM parameter values differed depending on the fitting method. A three-step non-linear least squares fitting approach, where D,f, and $D^{*}$ were estimated sequentially, generally yielded the lowest spatial and temporal variation. Solving all parameters simultaneously yielded the lowest residual between measured and fit data, however there was substantial spatial and temporal variability. Results obtained by Bayesian fitting had high spatial and temporal variability in addition to a large residual between measured and fit data. Simulations showed that all fitting methods can fit the IVIM data at signal to noise ratios >35, and that $D^{*}$ was the most challenging to accurately obtain. Overall, this study motivates use of a three-step non-linear least squares fitting strategy to quantify IVIM parameters in skeletal muscle.

## 1 Introduction

Measurement of blood flow to skeletal muscle provides insight into muscle metabolism, and activation in response to exercise. Non-invasive magnetic resonance imaging (MRI) methods to measure microvascular blood flow in skeletal muscle such as arterial spin labeling (ASL) and intra-voxel incoherent motion (IVIM) have been employed in a variety of muscles, including those in the leg (1–4), shoulder (5, 6), and lumbar spine (7–9). While ASL can quantify perfusion in physiologically relevant units, its spatial extent is somewhat limited and the perfusion response to stimuli such as exercise or induced ischemia dissipates quickly (10). Moreover, it remains difficult to quantify the low perfusion state of muscle at rest due to the limited signal-to-noise ratio (SNR) of ASL data.

Alternatively, IVIM is based on the incoherent translational motion of intravascular and intracellular water, and therefore is sensitive to the entire capillary blood volume within a voxel. Building off the theory of molecular diffusion, the motion of water in randomly oriented capillaries will also result in incoherent phase accrual, leading to signal decay in the presence of diffusion-encoding gradients (11, 12). Due to the difference in scale of motion in the intracellular (e.g., molecular diffusion, D) vs. intravascular (so called pseudo-diffusion, $D^{*}$) spaces, signal in intravascular space decays much more quickly than the intracellular component (13). The molecular and pseudo-diffusion coefficients, along with the volume fraction of the intravascular space (i.e., perfusion fraction, f) can be estimated by fitting data acquired at multiple diffusion encoding strengths to a bi-exponential model.

To estimate the IVIM parameters f,D, and $D^{*}$ from diffusion-weighted images, a variety of fitting strategies have been employed (14–16). However, in skeletal muscle, where resting perfusion is quite low, the rationale behind the choice of a particular analysis approach has not been clearly and methodically evaluated. Currently, there is not a single agreed upon analysis strategy, resulting in a lack of comparability of results across studies. Additionally, given that studies using IVIM to evaluate skeletal muscle perfusion may interrogate the response to exercise either acutely (same day) or over the course of some therapeutic intervention, it is also important to understand the repeatability of IVIM parameters in skeletal muscle at rest. Thus, the purpose of this work was to compare the estimates of IVIM parameters obtained via different fitting methods to ascertain the optimal strategy and normative values for muscles of the lumbar spine and to determine the variability of IVIM parameters across sessions. The findings of this study are essential for enhancing comparability across studies and establishing a consensus analysis strategy for future research.

## 2 Methods

### 2.1 Study design

This study was approved by the Institutional Review Board of the University of California, San Diego. Sixteen healthy people were recruited for the study after providing written informed consent. Participants were excluded if they had contraindications to MRI or had a history of low back pain. Individuals were instructed to avoid any physical activity on the day of the testing session. MR imaging was performed at 3T (MR750, GE Healthcare, Milwaukee, Wisconsin, USA) using a CTL phased-array spine coil for signal reception. The imaging protocol consisted of localizers, T1-weighted anatomical reference images, followed by collection of IVIM data while the subject rested in the supine position. Twelve participants returned for a second, identical visit, allowing for the evaluation of inter-session repeatability of the IVIM parameters.

### 2.2 Image acquisition

IVIM data were collected using an axial 2D, diffusion-weighted, spin-echo EPI sequence with: FOV = 192 × 192 mm^2^, slice thickness = 8 mm, 22 slices covering L1–S1, matrix = 128 × 128, TR/TE = 2,295/52.5 ms, flip angle = 90°, spatial-spectral fat saturation, number of repeats = 4, 3 directions of diffusion encoding with *b*-values = 0, 10, 20, 40, 70, 110, 160, 220, 300, 400, 500, 600, 700 s/mm^2^, and time = 344 s. The phase encoding direction was set to anterior-posterior to reduce the impact of any residual signal from subcutaneous fat in the tissue of interest.

### 2.3 Image analysis

**FIGURE 1:**
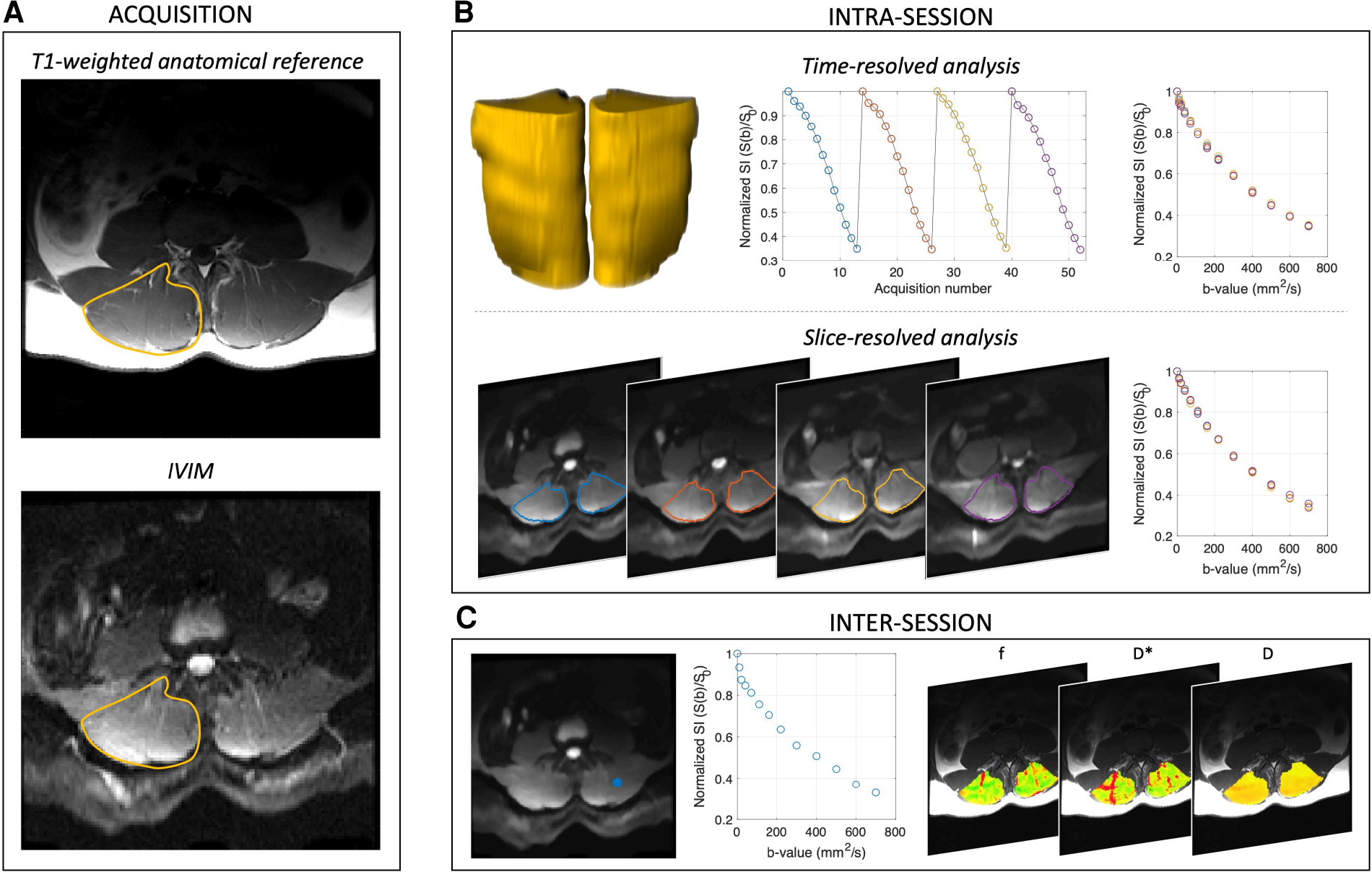
Acquisition and analysis overview. (**A**) Shows acquisition scheme, and the right paraspinal ROI is indicated in yellow on the T1-weighted anatomical reference image. (**B**) Intra-session analyses were performed on the time-resolved (yellow volumes, whole muscle ROI), spatially averaged (to assess temporal stability) and slice-averaged, time averaged data (to evaluate spatial consistency) separately. (**C**) Inter-session repeatability was evaluated by first computing the voxelwise IVIM parameter maps for $f,\;D^{*},\;D$, and ${fD}^{*}$, then averaging across the paraspinal ROI and comparing between scan sessions. Representative signal decay curve from a single voxel [blue dot in left panel of (**C**)] is shown in the middle panel of (**C**). In all cases, IVIM parameters were obtained by four different approaches including variably segmented, non-linear least squares fitting approaches and a Bayesian fitting method.

Image acquisition and analysis procedures are summarized in Figure 1. Specifically, the single IVIM acquisition described above was used for all sub-analyses, which are described in detail subsequently. Briefly, the four repeats were analyzed independently to evaluate intra-session temporal stability across the entire paraspinal muscle ROI. To assess intra-session spatial consistency the repeats were averaged across the entire paraspinal muscle ROI, and the mean diffusion-weighted signal in each slice was used for analysis. To assess Inter-session repeatability, repeated exams on separate days were compared in the subset of participants in which data were available.

#### 2.3.1 Preprocessing

Phase and distortion correction of the diffusion-weighted images was performed via TOPUP based on blip-up and blip-down calibration scans acquired at the end of the IVIM acquisition (17, 18). Denoising was conducted using a local principal component analysis filter (19). Rigid body motion correction over the dynamic image series was performed in AFNI (20).

#### 2.3.2 Image processing

Preprocessed data were imported into MATLAB (R2017a, Natick, MA), and subsequent analysis was performed using in-house written code, which can be found at https://github.com/ekenglund/Muscle_IVIM_analysis. First, a 2D 3 × 3 median filter was applied. IVIM data were then averaged over the three diffusion encoding directions for each *b*-value, yielding four series of low-to-high *b*-value images (one series per repeat). The four repeats were initially analyzed separately to assess temporal stability, then were averaged together prior to the calculation of slice-wise and voxelwise IVIM parameter maps (described below). SNR was calculated from a b = 0 s/mm^2^ image for each subject by dividing the mean of the signal of a region of interest (ROI) placed within the muscle by the standard deviation of the signal of an equally sized ROI placed outside of the body (21, 22).

#### 2.3.3 IVIM parameter fitting

For each analysis, IVIM parameters f, $D^{*}$, and D were obtained assuming bi-exponential signal decay as a function of *b*-value (13):

$$\frac{S(b)}{S_{0}}=f(e^{-{bD}^{*}})+(1-f)(e^{-bD})$$

where $S(b)/S_{0}$ is the measured diffusion-weighted data normalized by the non-diffusion weighted acquisition, f is the perfusion fraction, representing the relative fraction of signal from the intravascular vs. extravascular (i.e., intracellular) spaces and serving to weight the bi-exponential model by the two coefficients, $D^{*}$ the pseudo-diffusion coefficient, and D the diffusion coefficient. The product of f and $D^{*}\left(fD^{*}\right)$ was also calculated. Four separate approaches were evaluated:

- *1-step non-linear least squares (NLLS) fitting*; all three IVIM parameters were computed simultaneously using an NLLS fitting approach.
- *2-step NLLS fitting*; first *D* was determined, assuming that the effect of pseudo-diffusion is negligible at high *b*-values as $\frac{S(b>2000)}{S_{0}}=A\left(e^{-bD}\right)$ where A is an offset term. Once D was obtained, the value was fixed and all data were used to determine f and $D^{*}$ from the full bi-exponential equation via NLLS fitting.
- *3-step NLLS fitting*; first the natural log of the high *b*-value data were fit to a first order polynomial to determine D. This line was extrapolated to $b=0\;s/{mm}^{2}$ to obtain f, and finally the NLLS fit of the full bi-exponential model was used to calculate $D^{*}$.

*Bayesian fitting*; a Bayesian-probability based approach was used to estimate D,f, and $D^{*}$ based on the joint posterior probability as described in (23, 24). See supplemental methods for specific details of the Bayesian fitting analysis.

For all NLLS fitting approaches, fit constraints were held constant: f=[0-0.5]; $D^{*}=[1.5-500]\times 10^{-3}\;s/{mm}^{2}$; and $D=[0-2.5]\times 10^{-3}\;s/{mm}^{2}$. In the case of segmented fitting, the cutoff for diffusion-only signal decay (e.g., high b-value regime) was $>200\;s/{mm}^{2}$, and subsequently all *b*-values were used to compute $D^{*}$ (and f, if applicable). In addition to the obtained parameter values, the norm of the residual between the measured and fit data was recorded for each fitting method.

ROIs encompassing in the left and right erector spinae and multifidus (together referred to as paraspinal) muscles were manually defined on all slices of the T1-weighted images in Horos (v3.3.6, www.horosproject.org) and transferred to the IVIM data as previously described (25). In the final mask, voxels were excluded if noise or motion caused the signal intensity of the first diffusion-weighted acquisition to exceed the non-diffusion-weighted acquisition. Additionally, voxels were excluded if the solution for any of the voxelwise fits of the IVIM parameters was equal to the set fit constraint to prevent bias or overfitting of the signal to the IVIM equation.

#### 2.3.4 Defining the optimal fitting strategy

Two assumptions were made in order to define the optimal fitting strategy for IVIM data collected in skeletal muscle: (1) IVIM parameters are temporally constant in skeletal muscle at rest (26); (2) the variability of IVIM parameters between adjacent slices within a spinal segment in healthy paraspinal muscle is likely negligible due to its segmental innervation and function as an intervertebral stabilizer (27, 28). Based on these assumptions, we considered the optimal method as one that minimized the residual between the measured and fit data and had the least variability over time and between adjacent slices (e.g., intra-session repeatability). Variability was assessed by the within-subject coefficient of variation (WS-CV) (29).

#### 2.3.5 Intra-session temporal stability

Average signal intensity was computed across the entire paraspinal ROI for each *b*-value and repeat (Figure 1B), leading to four temporally-resolved signal decay curves per participant. Data were normalized by the non-diffusion weighted signal intensity, and IVIM parameters were computed via the four fitting methods for each repeat of the IVIM acquisition. The mean and standard deviation (SD) of $f,\;D^{*},\;D,\;{fD}^{*}$, and the squared residual norm were computed for each fitting method, and variability of IVIM parameters was assessed by the WS-CV.

#### 2.3.6 Intra-session spatial consistency

Diffusion weighted data were averaged across the four repeats, and within the ROI for each of the 22 slices, yielding one signal decay curve per slice. IVIM parameters were then computed with each fitting method for each slice (Figure 1B). Four slices spanning the superior and inferior margins of the vertebral body were assigned to each lumbar spine segment (L1–L5), and the variability within a spinal segment was evaluated. Mean and SD of IVIM parameters and mean squared residual norm for each fitting method were determined. Assuming that physiologically, perfusion and diffusion are relatively constant within a spinal segment (e.g., across all four slices located within L1), the WS-CV was computed for each of the five spinal segments as a metric of spatial consistency.

#### 2.3.7 Inter-session repeatability

For inter-session repeatability analysis, voxelwise parameter maps were computed within the defined paraspinal muscle ROI for the baseline and repeat scans using each of the four previously defined fitting methods (Figure 1C). Following voxelwise quantification of $f,\;D^{*},\;D$, and ${fD}^{*}$, IVIM parameter means and SD were computed over the entire paraspinal ROI as well as within the erector spinae and multifidus muscles separately, and results were compared between fitting methods. The SD of IVIM parameters for all voxels within the ROI was computed for both the baseline and repeat data to assess dispersion. The average percent of voxels included in the final ROI was also determined and compared between analysis methods. WS-CV was computed to assess the repeatability of ROI-averaged IVIM parameters across scan sessions.

### 2.4 Simulation

To understand the bias and precision of these fitting methods under relevant signal to noise ratio (SNR) conditions, a Monte Carlo based simulation was performed. First, a series of random combinations of $f,\;D^{*}$, and D were generated within the constraints f=[0–0.5]; $D^{*}=[1.5-500]\times 10^{-3}{\;mm}^{2}/s$; and $D=[0-2.5]\times 10^{-3}{\;mm}^{2}/s$. Then, the IVIM equation was solved for *S*(*b*)/*S*_0_ for the *b* values = [0, 10, 20, 40, 70, 110, 160, 220, 300, 400, 500, 600, 700] s/mm^2^ given f,D, and $D^{*}$. Gaussian noise was added to each voxel corresponding to SNR levels of 15, 20, 25, 30, 35, 40, 45, 50, 55, 60, 65, 70, 75, 80, yielding a total of 110,245 simulated IVIM experiments. For each noise condition, the synthetic signal was fit using the 1-Step NLLS, 2-Step NLLS, 3-Step NLLS, and Bayesian fitting approaches. An interrater correlation coefficient (2, 1) was calculated for each fitting method for each SNR level to assess the bias between fit parameters of f,D, and $D^{*}$ and the original values. ${fD}^{*}$ was not explicitly evaluated in the simulation model, as it the product of two o coefficients that were directly in the model. Therefore, its accuracy depends on the individual accuracy of these parameters.

### 2.5 Statistical analyses

Repeated measures one-way Analysis of Variance (ANOVA) tests were used to compare the IVIM parameter values between fitting methods for each analysis.

## 3 Results

Image artifacts precluded analysis in one subject, leaving 15 participants (mean ± SD, age = 44.4 ± 13.0 years, 8 male) for intra-session and 11 (42.5 ± 14.6 years, 6 male) for inter-session repeatability assessments. The average time between scan sessions was 76 ± 37 days. SNR of the images was 46.6 ± 10.6.

### 3.1 Intra-session temporal stability

**FIGURE 2:**
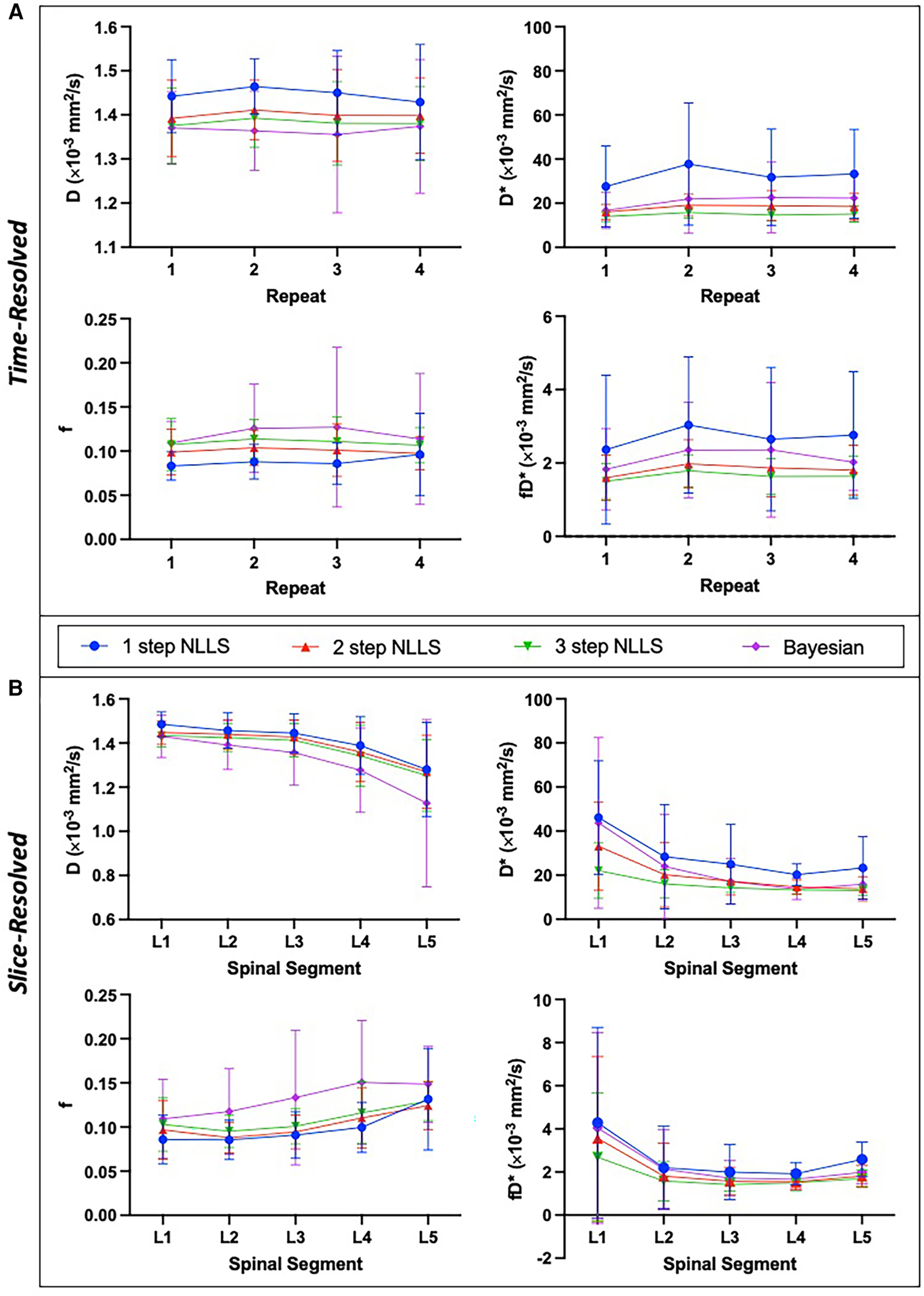
Summary of intra-session IVIM parameters showing mean and standard deviation across N=15 healthy individuals. IVIM parameters are shown for the temporally resolved (**A**) and slice-wise resolved (**B**) analyses for each fitting method. Slice-resolved analyses in (**B**) were evaluated on a slice-by-slice basis then averaged across slices and summarized here. In general, there was a significant difference between IVIM parameter values depending on the choice of fitting method (*p* < 0.0001 for both time-resolved and slice-resolved analyses on RM-ANOVA). The slice-wise analysis revealed a significant difference of IVIM parameters across the lumbar spine as well (*p* < 0.0001 for $f,\;D,\;D^{*}$ and *p* = 0.0002 for ${fD}^{*}$ on RM-ANOVA comparing individual parameters across spinal segments).

Averaged over the entire paraspinal muscle ROI, it was observed that overall, the choice of analysis method resulted in significantly different IVIM parameters (*p* < 0.0001 for all parameters, Figure 2A), and three-step fitting had the least temporal variability (Table 1). The average squared norm of the residual was highest for Bayesian fitting (0.0016) and was improved for all NLLS fitting methods. WS-CV was minimized for all IVIM parameters with the three-step fitting approach.

### 3.2 Intra-session spatial consistency

IVIM parameters calculated from slice-wise mean signal intensity, averaged within a spinal segment similarly differed based on the choice of analysis method (*p* < 0.0001), and additionally all parameters differed as a function of spinal segment (*p* < 0.0001) (Figure 2B). The mean residual was smallest when using one-step fitting (0.0007), however this led to substantial variation in the quantified parameters. The average WS-CV for one-step fitting was 24.2% for f, 45.2% for $D^{*}$, 5.9% for D, and 40.2% for ${fD}^{*}$. Three-step fitting had the least variability within a spinal segment. The mean WS-CV for three-step fitting was 15.4% for f, 24.5% for $D^{*}$, 4.2% for D, and 29.2% for $fD^{*}$ (Table 1). Regardless of fitting strategy, it was generally observed that across spinal segments, greater variability in IVIM parameters was observed in L1 and L5 compared to L2–L4 (data are included as Supporting Information, Supplementary Table S1 and Figure S1).

### 3.3 Inter-session repeatability

**FIGURE 3:**
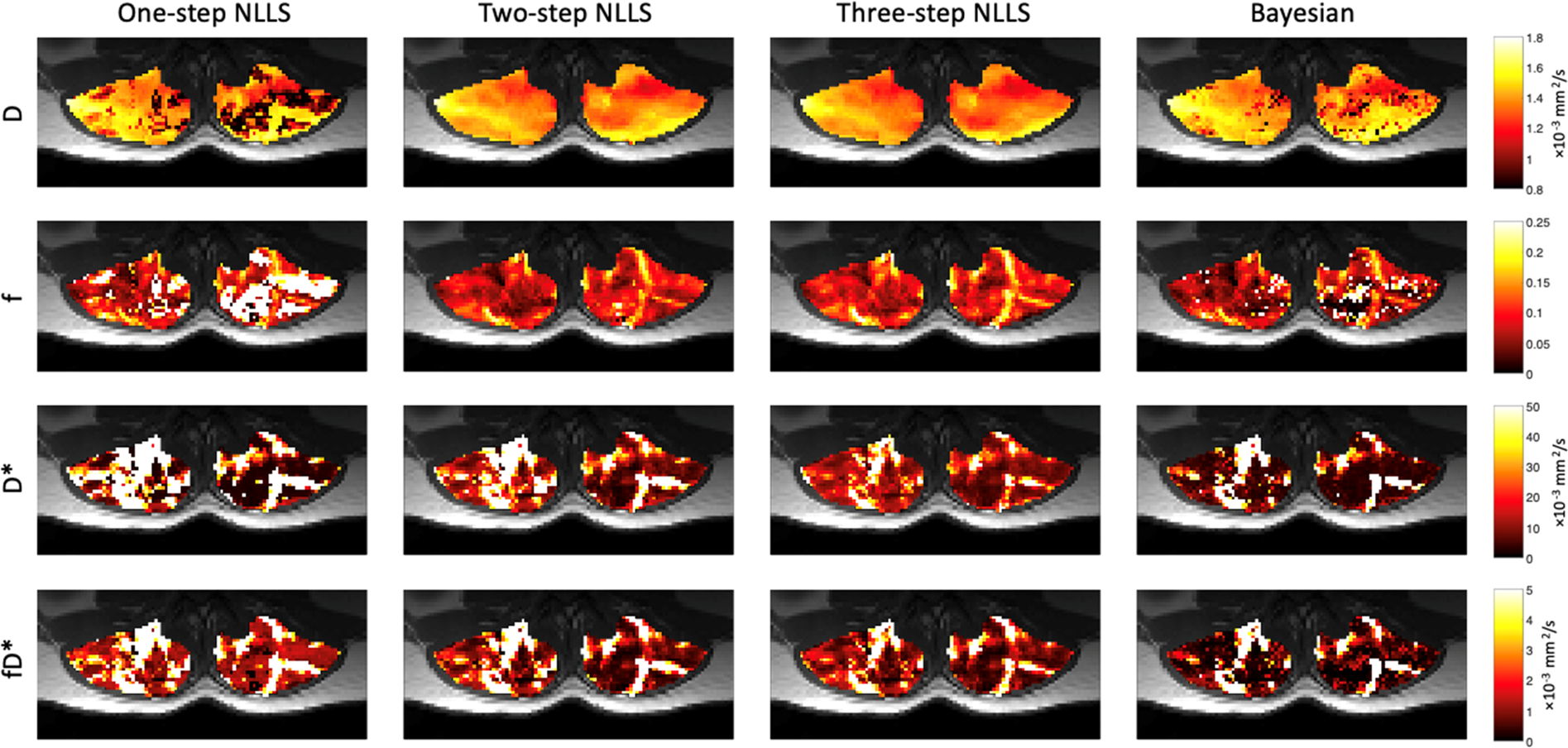
Representative IVIM parameter maps for each fitting strategy. In general, D was very spatially uniform across the paraspinal muscles. Relative anatomic contrast is observed in $f,\;D^{*}$, and $fD^{*}$ maps for the 2- and 3-step NLLS fitting approaches.

Figure 3 shows quantified IVIM parameter maps for a representative subject using each of the four fitting strategies. Comparing the two visits, when voxelwise IVIM parameters were averaged across the entire paraspinal muscle ROI, the WS-CV values were in general lower than those observed for the intra-session repeatability assessment (Table 1). Table 2 summarizes the results for the IVIM parameters at the first MRI scan session. In general, two- and three-step fitting methods yielded very similar results. One-step fitting led to higher f and $D^{*}$ values and lower D, and $D^{*}$ was significantly lower for Bayesian fitting. The spread of IVIM parameters (assessed by the mean SD across voxels) was generally lower for two-step and three-step fitting. The final mask comprised 62%, 72%, 71%, and 76% of voxels for one-, two-, three-step NLLS, and Bayesian fitting methods, respectively.

### 3.4 Simulation results and bias evaluation

**FIGURE 4:**
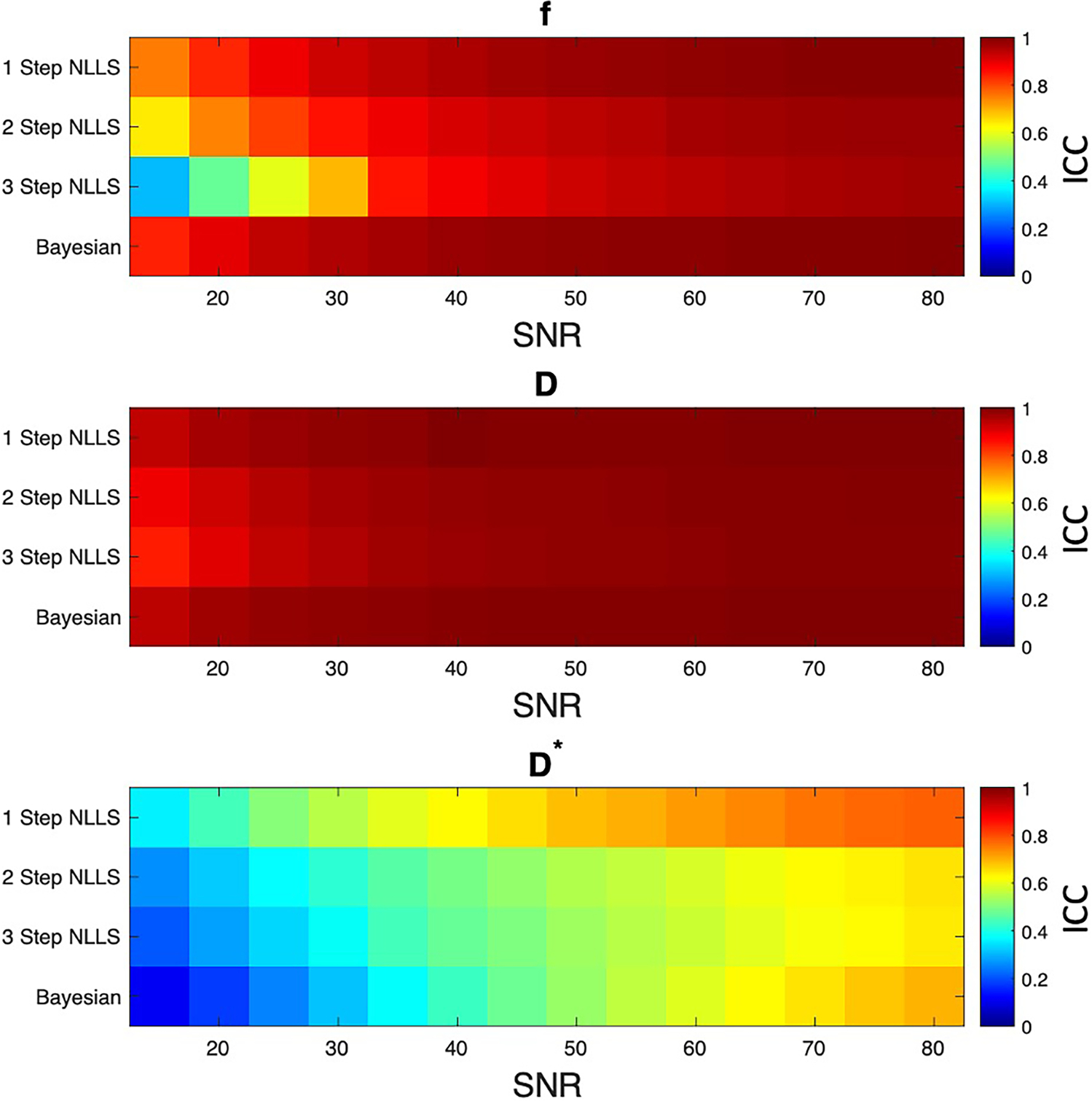
Results of simulation experiment. Ideal signal decay curves were generated from varied f,D, and $D^{*}$ values, then Gaussian noise was added to correspond to different levels of image SNR. Each fitting method was used to estimate IVIM parameters, and the interrater correlation coefficient (ICC) was computed between the simulated and fit parameter values. In general, 1 step non-linear least squares fitting had the least bias between simulated and fit parameters. The other fitting methods were in general agreement between simulated and fit data at SNR levels above 35.

110,245 different combinations of f,D, and $D^{*}$ were evaluated in this analysis. Overall, excellent agreement was found f and D for SNR levels over 35 for all fitting approaches (ICC > 0.8, Figure 4). The ability to fit $D^{*}$ was best for 1-Step NLLS fitting, and fair for 2-Step NLLS, 3-Step NLLS and Bayesian fitting.

## 4 Discussion

This study evaluated the variability of IVIM parameters in skeletal muscle at rest using a variety of fitting methods. With additional use of IVIM in healthy controls, in response to exercise or other interventions, or in patient populations, it is important to understand the typical IVIM parameter values in skeletal muscle at rest in healthy individuals, the repeatability of those parameters across scan sessions, and how fitting methods may influence those values. This methodical evaluation of the various fitting strategies demonstrated that the selected fitting strategy directly influences the calculation and interpretation of output IVIM parameters. Provided that our assumptions that skeletal muscle at rest has relatively temporally stable (over several minutes) and spatially consistent (within one spinal segment) perfusion and diffusion characteristics are valid, the optimal non-linear least squares fitting approach appears to be the segmented three-step fitting method as intra-session temporal and spatial variability was minimized and few voxels were excluded from the final mask.

The simulations performed herein echo the experimental results, finding that the one-step NLLS fitting method had the lowest bias between known and fit parameters, but that all fitting methods provide reasonable estimates of the IVIM parameters at SNR >35. Given that the average SNR of our experimental data was 46.6, we posit that there is negligible difference of bias or precision between fitting methods. A prior study by Damon defined the threshold for accurate diffusion measurements as 40 (30), in relative agreement with what was observed in our simulation. However, in general, image acquisition should be optimized to achieve high SNR in order to ensure accurate measurements of perfusion and restricted diffusion.

In addition to SNR, another important factor to consider for IVIM analysis in skeletal muscle is fat suppression. Both intra-muscular fat depots as well as subcutaneous fat can lead to potential errors IVIM. In this study, spatial-spectral fat suppression was used. Further, the low-bandwidth phase encoding direction was selected to be anterior to posterior, such that any unsuppressed signal from subcutaneous fat would alias posteriorly, outside of the anatomy of interest. Nevertheless, the seminal study by Cameron et al. (31) demonstrates that effective fat suppression is crucial for accurate measurement of diffusion parameters including IVIM. A conclusion from the Cameron, et al. study is that even with triple-fat suppressed data, there may still be reliability issues when estimating $D^{*}$. Both the Cameron et al. and this paper agree that $D^{*}$ is routinely the most difficult IVIM-based parameter to accurately estimate given currently available pulse sequence parameters and analysis approaches.

While one-step fitting minimized the residual between measured and fit data in both experimental and simulated data, it resulted in substantial temporal and spatial variability and the final mask contained the fewest voxels due to solutions occurring at the set boundary condition for at least one parameter from experimental data. Several prior studies have evaluated fitting methods for analysis of IVIM parameters (15, 16, 32, 33). In general, those studies have focused on the evaluation of IVIM parameters in cancerous tissues and have found, in agreement with the results herein, that parameter estimates for f,D, and $D^{*}$ differ depending on the fitting algorithm selected. A number of additional algorithms have been evaluated recently, including convolutional neural network based parameter estimation strategies (34). Overall, the results presented herein demonstrate the importance of considering the fitting method when comparing results across prior studies.

In general, it was observed that *D* had the least intra-session spatial and temporal variability, and the lowest inter-session variability of the quantified IVIM parameters. Perfusion fraction was stable with intra-session WS-CVs of ~15% and inter-session WS-CVs <10%. $D^{*}$ and $fD^{*}$ had higher variability, suggesting that it may be more difficult to detect the effect of an intervention with these parameters. However, prior studies have demonstrated changes in $D^{*}$ on the order of 100% (5–9) and as large as 289% (3) in response to acute bouts of exercise. Nevertheless, the variability and sensitivity of parameters needs to be taken into consideration when powering studies and interpreting results.

Comparing these results to prior studies, there has been a wide range of IVIM parameters reported in muscle (8, 9, 35). This variability may in part be due to the lack of standardization of fitting approach, as results herein show significant differences in parameter values depending on choice of analysis method. A recent study also evaluated the intra- and inter-session repeatability of IVIM parameters in tumor and muscle, finding improved repeatability with Bayesian fitting, however the mean values of IVIM parameters in muscle were not provided (36). Ranges for IVIM parameters in skeletal muscle have spanned 0.5 (37) to 2.19 (4) × 10^−3^ mm^2^/s for *D*; 0.03 (3) to 0.26 (37) for *f*; and 8 (38) to 287 (39) × 10^−3^ mm^2^/s for $D^{*}$.

Focusing on only investigations of the paraspinal muscles, a recent study by Federau, et al. examined the effect of lumbar extension exercise on IVIM metrics in controls and patients with adolescent idiopathic scoliosis (7). IVIM parameters were quantified via two-step fitting in the paraspinal muscles at rest in healthy volunteers were in reasonable agreement with those reported herein; $f,\;D^{*}$, and D: 0.10, 32 × 10^−3^ mm^2^/s, and 1.4 × 10^−3^ mm^2^/s, (right), and 0.8, 53 × 10^−3^ mm^2^/s, and 1.4 × 10^−3^ mm^2^/s (left). In addition to evaluating the IVIM parameters at rest, we have previously assessed the response to high intensity lumbar spine extension exercise in healthy individuals and people with low back pain using a three-step fitting approach (9). The response to varied exercise intensity was also evaluated in healthy individuals (8). Results of these prior studies showed that acute, targeted exercise is associated with an increase in IVIM parameters. Importantly, differences in IVIM parameters have been shown to be clinically relevant, for example, changes in D and $D^{*}$ in response to an acute exercise bout were shown to be highly predictive of reductions in disability and pain in response to an exercise-based rehabilitation program in individuals with low back pain, suggesting that this method may have utility in identifying individuals who may benefit from prescribed treatments (9). It also may help better understand the contribution of underlying microvascular impairments in various musculoskeletal conditions (7, 40).

Our results here and in a previous, independent study (41) suggest that diffusion characteristics differ in the paraspinal muscles as a function of spinal segment. The observation of changing mean diffusivity over the span of the lumbar spine may draw into question the assumption of spatial uniformity, however we note that spatial consistency was assumed within one spinal segment, not across the entire spine. Indeed, differences in IVIM parameters across the spinal segments are likely to reflect differences in structure and physiology of the paraspinal muscles from cranial to caudal, including differences in size (including the relative contribution of erector spinae vs. multifidus), tissue composition, and metabolic capacity (41–43). We also note that the IVIM parameters were generally more variable at L1 and L5, potentially due to respiratory motion at the superior extent and coil sensitivity at the inferior extent.

This study has several limitations. First, there is no gold standard to compare the experimental results. Simulations of various bi-exponential fitting approaches in the context of varied measurement noise have been completed previously (15), and were performed here to evaluate bias. In this study, we sought to primarily make use of the acquired data, based on assumptions regarding the underlying physiology to evaluate the variability. However, the lack of a true gold standard with which to compare the experimental data remains a limitation. In addition, we did not evaluate the choice of cutoff value for the high *b*-value regime (44), which may be important when analyzing IVIM parameters in response to a physiologic challenge such as exercise, nor did we evaluate the influence of pre-processing steps, such as applying a median filter. Last, we did not evaluate any variation in the cutoff ranges of IVIM parameters, but instead chose to use a wide range of physiologically plausible values for f,D, and $D^{*}$. Given that the mean D was less than 1.4 × 10^−3^ mm^2^/s, the minimum $D^{*}$ was set to 1.5 × 10^−3^ mm^2^/s. Overlapping ranges between D and $D^{*}$ could lead to issues with fit convergence, though this was not practically observed.

### 4.1 Conclusion

Overall, this study compares repeatability of IVIM parameters in skeletal muscle at rest using a variety of bi-exponential fitting strategies and helps to define normal values of IVIM parameters in muscles of the lumbar spine at rest in healthy adult volunteers. The three-step NLLS fitting approach yielded results with the least temporal and spatial variability, low inter-session variability, and a large inclusion rate of voxels in the final mask. Future studies evaluating muscles of the lumbar spine in response to acute bouts of exercise, pathology, or long-term rehabilitation therapy may provide additional insight into the physiologic underpinnings of the IVIM parameters in skeletal muscle.

## Supplementary Material

Supplementary Material

## Figures and Tables

**TABLE 1:** Summary of intra-session and inter-session variability. WS-CV is within-subject coefficient of variation.

Intra-session: Time-resolved analysis	WS-CV (%)				Norm of fit residuals
	*f*	D*	*D*	fD*	
1-step NLLS	21.5	51.5	4.1	42.1	0.0007
2-step NLLS	14.4	27.7	2.0	28.1	0.0010
3-step NLLS	13.7	17.4	1.8	22.1	0.0011
Bayesian	36.0	48.7	6.7	36.5	0.0016
Intra-session: slice-resolved analysis (averaged across all spinal segments)	Mean (SD) WS-CV (%) across spinal segments				Norm of fit residuals
	*f*	D*	*D*	fD*	
1-step NLLS	24.2 (8.7)	45.2 (15.5)	5.9 (3.1)	40.2 (15.6)	0.0007
2-step NLLS	17.1 (3.8)	34.3 (17.4)	4.4 (1.5)	35.3 (14.8)	0.0010
3-step NLLS	15.4 (2.3)	24.5 (12.7)	4.2 (1.3)	29.2 (12.6)	0.0011
Bayesian	37.9 (6.7)	49.4 (14.2)	9.7 (1.7)	36.5 (11.3)	0.0013
Inter-session	WS-CV (%)				Norm of fit residuals
	*f*	D*	*D*	fD*	
1-step NLLS	7.9	13.1	1.4	15.6	0.0026
2-step NLLS	9.7	14.4	1.7	17.7	0.0032
3-step NLLS	8.9	12.7	1.6	16.9	0.0036
Bayesian	9.7	18.4	1.6	21.2	0.0100

**TABLE 2:** Summary of mean (standard deviation) IVIM parameter values across the paraspinal ROI at visit 1. The dispersion of parameter values in the voxelwise map is presented on bottom.

Visit 1—full paraspinal Parameter mean (SD)	*f*	D* (×10^−3^ mm^2^/s)	D (×10^−3^ mm^2^/s)	fD*(×10^−3^ mm^2^/s)
1-step NLLS	0.15 (0.02)	40.7 (15.6)	1.33 (0.08)	4.66 (2.14)
2-step NLLS	0.11 (0.02)	32.4 (10.4)	1.37 (0.09)	3.53 (1.55)
3-step NLLS	0.11 (0.02)	28.4 (6.8)	1.36 (0.08)	2.95 (1.17)
Bayesian	0.12 (0.02)	17.6 (7.1)	1.35 (0.08)	2.46 (1.21)
Visit 1—erector spinae muscle parameter mean (SD)	*f*	D* (×10^−3^ mm^2^/s)	D (×10^−3^ mm^2^/s)	fD*(×10^−3^ mm^2^/s)
1-step NLLS	0.15 (0.01)	42.2 (16.1)	1.35 (0.07)	4.64 (2.23)
2-step NLLS	0.11 (0.02)	34.2 (11.2)	1.39 (0.08)	3.58 (1.68)
3-step NLLS	0.11 (0.02)	30.3 (7.5)	1.38 (0.07)	3.02 (1.28)
Bayesian	0.12 (0.02)	17.9 (7.9)	1.38 (0.07)	2.36 (1.30)
Visit 1—multifidus muscle parameter mean (SD)	*f*	D* (×10^−3^ mm^2^/s)	D (×10^−3^ mm^2^/s)	fD*(×10^−3^ mm^2^/s)
1-step NLLS	0.17 (0.03)	35.7 (14.8)	1.25 (0.11)	4.65 (2.16)
2-step NLLS	0.12 (0.03)	27.2 (10.0)	1.31 (0.12)	3.33 (1.48)
3-step NLLS	0.12 (0.03)	22.7 (6.6)	1.30 (0.12)	2.70 (1.03)
Bayesian	0.14 (0.03)	16.7 (6.0)	1.29 (0.12)	2.68 (1.51)
Visit 1—mean (SD) of the dispersion across full paraspinal muscle ROI	*f*	D* (×10^−3^ mm^2^/s)	D (×10^−3^ mm^2^/s)	fD*(×10^−3^ mm^2^/s)
1-step NLLS	0.12 (0.01)	63.3 (15.4)	0.27 (0.04)	8.69 (3.22)
2-step NLLS	0.07 (0.01)	56.3 (16.1)	0.21 (0.06)	7.09 (2.96)
3-step NLLS	0.07 (0.01)	56.3 (11.9)	0.21 (0.06)	5.80 (2.56)
Bayesian	0.12 (0.02)	31.4 (11.0)	0.27 (0.06)	6.42 (3.09)

## Data Availability

The raw data supporting the conclusions of this article will be made available by the authors, without undue reservation.

## References

[1] Raynaud JS, Duteil S, Vaughan JT, Hennel F, Wary C, Leroy-Willig A, Determination of skeletal muscle perfusion using arterial spin labeling NMRI: validation by comparison with venous occlusion plethysmography. Magn Reson Med. (2001) 46(2):305–11. doi: 10.1002/mrm.1192 11477634

[2] Suo S, Zhang L, Tang H, Ni Q, Li S, Mao H, Evaluation of skeletal muscle microvascular perfusion of lower extremities by cardiovascular magnetic resonance arterial spin labeling, blood oxygenation level-dependent, and intravoxel incoherent motion techniques. J Cardiovasc Magn Reson. (2018) 20(1):18. doi: 10.1186/s12968-018-0441-3 29551091 PMC5858129

[3] Filli L, Boss A, Wurnig MC, Kenkel D, Andreisek G, Guggenberger R. Dynamic intravoxel incoherent motion imaging of skeletal muscle at rest and after exercise. NMR Biomed. (2015) 28(2):240–6. doi: 10.1002/nbm.3245 25521711

[4] Adelnia F, Shardell M, Bergeron CM, Fishbein KW, Spencer RG, Ferrucci L, Diffusion-weighted MRI with intravoxel incoherent motion modeling for assessment of muscle perfusion in the thigh during post-exercise hyperemia in younger and older adults. NMR Biomed. (2019) 32(5):e4072. doi: 10.1002/nbm.4072 30861224 PMC6530599

[5] Nguyen A, Ledoux JB, Omoumi P, Becce F, Forget J, Federau C. Application of intravoxel incoherent motion perfusion imaging to shoulder muscles after a lift-off test of varying duration. NMR Biomed. (2016) 29(1):66–73. doi: 10.1002/nbm.3449 26684052

[6] Nguyen A, Ledoux JB, Omoumi P, Becce F, Forget J, Federau C. Selective microvascular muscle perfusion imaging in the shoulder with intravoxel incoherent motion (IVIM). Magn Reson Imaging. (2017) 35:91–7. doi: 10.1016/j.mri.2016.08.005 27576020

[7] Federau C, Kroismayr D, Dyer L, Farshad M, Pfirrmann C. Demonstration of asymmetric muscle perfusion of the back after exercise in patients with adolescent idiopathic scoliosis using intravoxel incoherent motion (IVIM) MRI. NMR Biomed. (2020) 33(3):e4194. doi: 10.1002/nbm.4194 31815323

[8] Englund EK, Berry DB, Behun JJ, Ward SR, Frank LR, Shahidi B. IVIM Imaging of paraspinal muscles following moderate and high-intensity exercise in healthy individuals. Front Rehabil Sci. (2022) 3. doi: 10.3389/fresc.2022.910068 PMC9365030 35959464

[9] Shahidi B, Behun JJ, Berry DB, Raiszadeh K, Englund EK. Intravoxel incoherent motion imaging predicts exercise-based rehabilitation response in individuals with low back pain. NMR Biomed. (2021) 34(12):e4595. doi: 10.1002/nbm.4595 34327758

[10] Englund EK, Rodgers ZB, Langham MC, Mohler ER 3rd, Floyd TF, Wehrli FW. Simultaneous measurement of macro- and microvascular blood flow and oxygen saturation for quantification of muscle oxygen consumption. Magn Reson Med. (2018) 79(2):846–55. doi: 10.1002/mrm.26744 28497497 PMC5681899

[11] Le Bihan D, Breton E, Lallemand D, Aubin ML, Vignaud J, Laval-Jeantet M. Separation of diffusion and perfusion in intravoxel incoherent motion MR imaging. Radiology. (1988) 168(2):497–505. doi: 10.1148/radiology.168.2.3393671 3393671

[12] Le Bihan D, Turner R. The capillary network: a link between IVIM and classical perfusion. Magn Reson Med. (1992) 27(1):171–8. doi: 10.1002/mrm.1910270116 1435202

[13] Le Bihan D What can we see with IVIM MRI? Neuroimage. (2019) 187:56–67. doi: 10.1016/j.neuroimage.2017.12.062 29277647

[14] Meeus EM, Novak J, Withey SB, Zarinabad N, Dehghani H, Peet AC. Evaluation of intravoxel incoherent motion fitting methods in low-perfused tissue. J Magn Reson Imaging. (2017) 45(5):1325–34. doi: 10.1002/jmri.25411 27545824 PMC5412931

[15] Cho GY, Moy L, Zhang JL, Baete S, Lattanzi R, Moccaldi M, Comparison of fitting methods and b-value sampling strategies for intravoxel incoherent motion in breast cancer. Magn Reson Med. (2015) 74(4):1077–85. doi: 10.1002/mrm.25484 25302780 PMC4439397

[16] Gurney-Champion OJ, Klaassen R, Froeling M, Barbieri S, Stoker J, Engelbrecht MRW, Comparison of six fit algorithms for the intra-voxel incoherent motion model of diffusion-weighted magnetic resonance imaging data of pancreatic cancer patients. PLoS One. (2018) 13(4):e0194590. doi: 10.1371/journal.pone.0194590 29617445 PMC5884505

[17] Andersson JL, Skare S, Ashburner J. How to correct susceptibility distortions in spin-echo echo-planar images: application to diffusion tensor imaging. Neuroimage. (2003) 20(2):870–88. doi: 10.1016/S1053-8119(03)00336-7 14568458

[18] Smith SM, Jenkinson M, Woolrich MW, Beckmann CF, Behrens TE, Johansen-Berg H, Advances in functional and structural MR image analysis and implementation as FSL. Neuroimage. (2004) 23(Suppl 1):S208–19. doi: 10.1016/j.neuroimage.2004.07.051 15501092

[19] Manjon JV, Coupe P, Concha L, Buades A, Collins DL, Robles M. Diffusion weighted image denoising using overcomplete local PCA. PLoS One. (2013) 8(9):e73021. doi: 10.1371/journal.pone.0073021 24019889 PMC3760829

[20] Cox RW. AFNI: software for analysis and visualization of functional magnetic resonance neuroimages. Comput Biomed Res. (1996) 29(3):162–73. doi: 10.1006/cbmr.1996.0014 8812068

[21] Henkelman RM. Measurement of signal intensities in the presence of noise in MR images. Med Phys. (1985) 12(2):232–3. doi: 10.1118/1.595711 4000083

[22] Kaufman L, Kramer DM, Crooks LE, Ortendahl DA. Measuring signal-to-noise ratios in MR imaging. Radiology. (1989) 173(1):265–7. doi: 10.1148/radiology.173.1.2781018 2781018

[23] Gustafsson O, Montelius M, Starck G, Ljungberg M. Impact of prior distributions and central tendency measures on Bayesian intravoxel incoherent motion model fitting. Magn Reson Med. (2018) 79(3):1674–83. doi: 10.1002/mrm.26783 28626964

[24] Jalnefjord O, Andersson M, Montelius M, Starck G, Elf AK, Johanson V, Comparison of methods for estimation of the intravoxel incoherent motion (IVIM) diffusion coefficient (D) and perfusion fraction (f). MAGMA. (2018) 31(6):715–23. doi: 10.1007/s10334-018-0697-5 30116979

[25] Berry DB, Padwal J, Johnson S, Parra CL, Ward SR, Shahidi B. Methodological considerations in region of interest definitions for paraspinal muscles in axial MRIs of the lumbar spine. BMC Musculoskelet Disord. (2018) 19(1):135. doi: 10.1186/s12891-018-2059-x 29734942 PMC5938809

[26] Englund EK, Reiter DA, Shahidi B, Sigmund EE. Intravoxel incoherent motion magnetic resonance imaging in skeletal muscle: review and future directions. J Magn Reson Imaging. (2022) 55(4):988–1012. doi: 10.1002/jmri.27875 34390617 PMC8841570

[27] Kalimo H, Rantanen J, Viljanen T, Einola S. Lumbar muscles: structure and function. Ann Med. (1989) 21(5):353–9. doi: 10.3109/07853898909149220 2532525

[28] Ward SR, Kim CW, Eng CM, Gottschalk L, Tomiya A, Garfin SR, Architectural analysis and intraoperative measurements demonstrate the unique design of the multifidus muscle for lumbar spine stability. J Bone Joint Surg Am. (2009) 91(1):176–85. doi: 10.2106/JBJS.G.01311 19122093 PMC2663324

[29] Bland JM, Altman DG. Measurement error proportional to the mean. Br Med J. (1996) 313(7049):106. doi: 10.1136/bmj.313.7049.106 8688716 PMC2351517

[30] Damon BM. Effects of image noise in muscle diffusion tensor (DT)-MRI assessed using numerical simulations. Magn Reson Med. (2008) 60(4):934–44. doi: 10.1002/mrm.21707 18816814 PMC2570042

[31] Cameron D, Bouhrara M, Reiter DA, Fishbein KW, Choi S, Bergeron CM, The effect of noise and lipid signals on determination of Gaussian and non-Gaussian diffusion parameters in skeletal muscle. NMR Biomed. (2017) 30(7):e3718. doi: 10.1002/nbm.3718 PMC5876728 28383778

[32] Suo S, Lin N, Wang H, Zhang L, Wang R, Zhang S, Intravoxel incoherent motion diffusion-weighted MR imaging of breast cancer at 3.0 tesla: comparison of different curve-fitting methods. J Magn Reson Imaging. (2015) 42(2):362–70. doi: 10.1002/jmri.24799 25407944

[33] Fusco R, Sansone M, Petrillo A. A comparison of fitting algorithms for diffusion-weighted MRI data analysis using an intravoxel incoherent motion model. MAGMA. (2017) 30(2):113–20. doi: 10.1007/s10334-016-0591-y 27670762

[34] Huang HM. An unsupervised convolutional neural network method for estimation of intravoxel incoherent motion parameters. Phys Med Biol. (2022) 67(21). doi: 10.1088/1361-6560/ac9a1f 36228623

[35] Englund EK, Reiter DA, Shahidi B, Sigmund EE. Intravoxel incoherent motion magnetic resonance imaging in skeletal muscle: review and future Directions. J Magn Reson Imaging. (2021) 55(4):988–1012. doi: 10.1002/jmri.27875 34390617 PMC8841570

[36] Koopman T, Martens R, Gurney-Champion OJ, Yaqub M, Lavini C, de Graaf P, Repeatability of IVIM biomarkers from diffusion-weighted MRI in head and neck: Bayesian probability versus neural network. Magn Reson Med. (2021) 85(6):3394–402. doi: 10.1002/mrm.28671 33501657 PMC7986193

[37] Becker AS, Wurnig MC, Finkenstaedt T, Boss A. Non-parametric intravoxel incoherent motion analysis of the thyroid gland. Heliyon. (2017) 3(1):e00239. doi: 10.1016/j.heliyon.2017.e00239 28180186 PMC5288302

[38] Phi Van VD, Becker AS, Ciritsis A, Reiner CS, Boss A. Intravoxel incoherent motion analysis of abdominal organs: application of simultaneous multislice acquisition. Invest Radiol. (2018) 53(3):179–85. doi: 10.1097/RLI.0000000000000426 29112516

[39] Xu XQ, Choi YJ, Sung YS, Yoon RG, Jang SW, Park JE, Intravoxel incoherent motion MR imaging in the head and neck: correlation with dynamic contrast-enhanced MR imaging and diffusion-weighted imaging. Korean J Radiol. (2016) 17(5):641–9. doi: 10.3348/kjr.2016.17.5.641 27587952 PMC5007390

[40] Sigmund EE, Baete SH, Luo T, Patel K, Wang D, Rossi I, MRI assessment of the thigh musculature in dermatomyositis and healthy subjects using diffusion tensor imaging, intravoxel incoherent motion and dynamic DTI. Eur Radiol. (2018) 28(12):5304–15. doi: 10.1007/s00330-018-5458-3 29869178 PMC11980643

[41] Berry DB, Rodriguez-Soto AE, Englund EK, Shahidi B, Parra C, Frank LR, Multiparametric MRI characterization of level dependent differences in lumbar muscle size, quality, and microstructure. JOR Spine. (2020) 3(2):e1079. doi: 10.1002/jsp2.1079 32613159 PMC7323468

[42] Agten A, Stevens S, Verbrugghe J, Eijnde BO, Timmermans A, Vandenabeele F. The lumbar multifidus is characterised by larger type I muscle fibres compared to the erector spinae. Anat Cell Biol. (2020) 53(2):143–50. doi: 10.5115/acb.20.009 32647082 PMC7343561

[43] Chen P, Zhou Z, Sun L, Yu X, Li K, Li J, Quantitative multi-parameter assessment of age- and gender-related variation of back extensor muscles in healthy adults using Dixon MR imaging. Eur Radiol. (2023) 34(1):69–79. doi: 10.1007/s00330-023-09954-w 37537425

[44] Wurnig MC, Donati OF, Ulbrich E, Filli L, Kenkel D, Thoeny HC, Systematic analysis of the intravoxel incoherent motion threshold separating perfusion and diffusion effects: proposal of a standardized algorithm. Magn Reson Med. (2015) 74(5):1414–22. doi: 10.1002/mrm.25506 25360990

