# Supplementary material for: Assessment of fitting methods and variability of IVIM parameters in muscles of the lumbar spine at rest
Source: Front Musculoskelet Disord. Author manuscript; Available in PMC 2024 Aug 12. (PMC11318298; doi:10.3389/fmscd.2024.1386276)
Supplement: Supplementary Material [file NIHMS2008421-supplement-Supplementary_Material.docx]

Supplemental Material.

Supplemental Methods:

Specific details for Bayesian fitting include:

its = 1e4;

rician = false;

prior = {'flat','lognorm','lognorm','flat'};

burns = 2000;

meanonly = false;

| **Table S1.** Within-subject coefficient of variation of slice-wise resolved IVIM parameters for each spinal segment. | | | | |
| --- | --- | --- | --- | --- |
| **Intra-session (Slice-resolved)**  **WS-CV (%)** | | | | |
|  | f | D* | D | fD* |
| *L1* | | | | |
| 1-step NLLS | 21.9 | 72.6 | 5.8 | 67.9 |
| 2-step NLLS | 21.1 | 63.5 | 6.1 | 59.9 |
| 3-step NLLS | 17.0 | 45.9 | 5.2 | 49.3 |
| Bayesian | 47.7 | 73.2 | 11.4 | 53.7 |
| *L2* | | | | |
| 1-step NLLS | 20.7 | 39.6 | 2.8 | 34.6 |
| 2-step NLLS | 14.6 | 27.6 | 2.5 | 28.6 |
| 3-step NLLS | 13.7 | 20.3 | 2.5 | 24.7 |
| Bayesian | 38.9 | 46.2 | 6.8 | 36.9 |
| *L3* | | | | |
| 1-step NLLS | 26.1 | 38.4 | 5.1 | 30.9 |
| 2-step NLLS | 21.0 | 33.8 | 4.1 | 29.7 |
| 3-step NLLS | 17.7 | 17.3 | 4.1 | 20.1 |
| Bayesian | 39.5 | 40.6 | 10.3 | 27.1 |
| *L4* | | | | |
| 1-step NLLS | 14.5 | 34.4 | 4.5 | 31.7 |
| 2-step NLLS | 12.6 | 17.3 | 3.4 | 21.3 |
| 3-step NLLS | 12.3 | 13.6 | 3.4 | 18.7 |
| Bayesian | 33.3 | 36.9 | 9.6 | 25.4 |
| *L5* | | | | |
| 1-step NLLS | 37.9 | 41.2 | 11.1 | 35.8 |
| 2-step NLLS | 16.3 | 29.3 | 5.8 | 37.1 |
| 3-step NLLS | 16.1 | 25.6 | 5.8 | 33.2 |
| Bayesian | 30.2 | 49.8 | 10.3 | 39.3 |

**Figure S1.** Slice-resolved results: The mean and standard deviation of parameter values across all healthy participants as a function of slice within spinal segment.
